# Does sub‐cortical PI‐2620 tau PET signal reflect Alzheimer's disease status?

**DOI:** 10.1002/alz70856_104171

**Published:** 2025-12-25

**Authors:** Shuo Ni, Jiaxin Yue, Yonggand Shi

**Affiliations:** ^1^ University of Southern California, Los Angeles, CA, USA; ^2^ Stevens Neuroimaging and Informatics Institute, Keck School of Medicine, University of Southern California, Los Angeles, CA, USA; ^3^ Department of Neurology, Stevens Neuroimaging and Informatics Institute, Keck School of Medicine, University of Southern California, Los Angeles, CA, USA

## Abstract

**Background:**

The first‐generation tau PET tracer AV‐1451 has been widely used to study tau pathology in Alzheimer's disease (AD), but it's high off‐target binding in sub‐cortical structures has limited our ability to examine possible tau pathology in these structures. With the increasing popularity of second‐generation tau PET tracers such as PI‐2620 and MK‐6240 that exhibit low off‐target binding signals in sub‐cortical structures, it is important to examine whether their signals can be useful in evaluating the severity of tau pathology of sub‐cortical structures in AD.

**Method:**

This study utilized tau PET data based on the PI‐2620 tracer from the Health and Aging Brain Study: Health Disparities (HABS‐HD). Overall, data used in this study are from 1,492 participants with both T1‐weighted MRI and tau PET imaging, along with the available clinical and cognitive metrics such as the Consensus Diagnosis (CDX). The cohort included 1097 cognitively unimpaired (CU) and 395 cognitively impaired (CI) participants, which are comprised of 479 Black, 447 Hispanic, and 566 non‐Hispanic White subjects. Subcortical regions of interest included key structures from both the left and right hemispheres including the thalamus, caudate, putamen, pallidum, hippocampus, amygdala, and accumbens. Automated segmentation maps were generated from T1‐weighted MRI using FreeSurfer and applied to the corresponding PET images to calculate mean standard uptake value ratio (SUVR) values for each subcortical region.

**Result:**

Significant differences in tau SUVR values were observed in the caudate (*p*‐value < 1e‐2), hippocampus (*p*‐value < 1e‐2), and amygdala (*p*‐value < 1e‐4) across diagnostic categories in the entire cohort. Ethnic group analyses for CU and CI subjects revealed statistically significant differences in different sub‐cortical regions. Among non‐Hispanic White participants, differences were significant in the putamen (*p*‐value < 1e‐2), hippocampus (*p*‐value < 1e‐2), and amygdala (*p*‐value < 1e‐5). Significant differences were observed in the caudate (*p*‐value < 1e‐2) for Black participants, while the amygdala showed statistically significant differences (*p*‐value < 5e‐2) for Hispanic participants.

**Conclusion:**

Ethnicity plays an important role on sub‐cortical tau PET signal for CU and CI, especially for non‐Hispanic White in the amygdala area.

**Funding Sources**: RF1AG077578, RF1AG064584, R01EB022744, U19AG078109, P30AG066530.

